# Activation of the viral sensor oligoadenylate synthetase 2 (*Oas2*) prevents pregnancy-driven mammary cancer metastases

**DOI:** 10.1186/s13058-022-01525-z

**Published:** 2022-05-03

**Authors:** Wing-Hong Jonathan Ho, Andrew M. K. Law, Etienne Masle-Farquhar, Lesley E. Castillo, Amanda Mawson, Moira K. O’Bryan, Christopher C. Goodnow, David Gallego-Ortega, Samantha R. Oakes, Christopher J. Ormandy

**Affiliations:** 1 Garvan Institute of Medical Research, 384 Victoria Street, Darlinghurst Sydney, NSW 2010 Australia; 2 St. Vincent’s Clinical School, St. Vincent’s Hospital, UNSW Sydney, Kensington, NSW Australia; 3 The School of BioSciences and Bio21 Institute, Faculty of Science, The University of Melbourne, Parkville, Melbourne, VIC 3010 Australia; 4 Cellular Genomics Futures Institute, UNSW Sydney, Kensington, NSW Australia; 5 Present Address: School of Biomedical Engineering, Faculty of Engineering and Information Technology, University of Technology Sydney, 81 Broadway, Ultimo Sydney, NSW 2007 Australia; 6 Present Address: National Breast Cancer Foundation Level 7, 50 Margaret Street, Sydney, NSW 2001 Australia

**Keywords:** OAS2, Interferon, Immunotherapy, Breast, Mammary, Cancer

## Abstract

**Background:**

The interferon response can influence the primary and metastatic activity of breast cancers and can interact with checkpoint immunotherapy to modulate its effects. Using *N*-ethyl-*N*-nitrosourea mutagenesis, we found a mouse with an activating mutation in oligoadenylate synthetase 2 (*Oas2*), a sensor of viral double stranded RNA, that resulted in an interferon response and prevented lactation in otherwise healthy mice.

**Methods:**

To determine if sole activation of *Oas2* could alter the course of mammary cancer, we combined the *Oas2* mutation with the *MMTV-PyMT* oncogene model of breast cancer and examined disease progression and the effects of checkpoint immunotherapy using Kaplan–Meier survival analysis with immunohistochemistry and flow cytometry.

**Results:**

*Oas2* mutation prevented pregnancy from increasing metastases to lung. Checkpoint immunotherapy with antibodies against programmed death-ligand 1 was more effective when the *Oas2* mutation was present.

**Conclusions:**

These data establish OAS2 as a therapeutic target for agents designed to reduce metastases and increase the effectiveness of checkpoint immunotherapy.

**Supplementary Information:**

The online version contains supplementary material available at 10.1186/s13058-022-01525-z.

## Background

The interferon response can influence the primary and metastatic activity of breast cancers [1], and can interact with checkpoint immunotherapy to modulate its effects [2]. Although systemic interferon delivery for cancer produced responses in tumors, it was limited by severe adverse events and has been superseded by newer therapies [3]. To circumvent some of these challenges, intratumor injection of agents that induce an interferon response are currently being trialed in a large number of cancer settings and among these agents are derivatives of polyinosinic–polycytidylic acid (polyIC) [4]. In mice, polyIC greatly improved the ability of anti-programmed death-ligand 1 (PD-L1) therapy to clear or reduce melanomas and cancers of the lung and colon, and this effect was dependent on type 1 interferons [5].

PolyIC and its degradation products mimic viral RNA, key pathogen-associated molecular-patterns displayed during viral replication. PolyIC binds to and activates toll-like receptor 3 (TLR3), a pathogen-associated molecular pattern receptor located on the cell membrane and within endosomes. This generates a number of immune cell effects, such as an interferon response and innate immune cell activation, the latter effect responsible for the use of polyIC as a vaccine adjuvant [6].

There is, however, another family of pathogen-associated molecular pattern receptors that recognize double-stranded RNA. The OAS family of proteins are potent inducers of the interferon response [7]. They are pathogen-associated molecular pattern receptors that bind double-stranded RNA in the cytoplasm. These proteins respond by synthesizing 2′–5′-linked RNA from ATP without a template [8]. This in turn binds to and activates latent ribonuclease (RNaseL), causing it to degrade viral RNA and host ribosomal RNA, so disrupting the viral life cycle. Activation of OAS-RNaseL also promotes apoptosis and provokes a robust interferon response, both via poorly understood mechanisms that involve additional detection of RNaseL-degraded RNA by the RIG1-like pathogen recognition receptors and possibly direct interaction of OAS and RNaseL with the mitochondria [9]. The OAS family is routinely ignored by most studies using polyIC, which focus on the action of TLR3. The potential of the OAS family as therapeutic targets in oncology has been ignored because the effects of polyIC are attributed to TLR3, and no effective way to separate the actions of OAS and TLR3 was available.

There is some evidence that RNaseL may be involved in the etiology and progression of cancer. Attention has focused on RNaseL, the down-stream effector of the OAS proteins, activation of which predicted the outcomes in virally induced cancers. An association with RNaseL allelic variation and the risk of human papilloma virus-driven cancers of the cervix and head was demonstrated. Unexpectedly, this study also found an association with cancers that do not show a strong viral etiology such as breast [10]. Activation of RNaseL may occur naturally in cancers, hypothesized to be driven by the recognition of double-stranded RNA species by OAS proteins produced from viral sequences integrated in the genome. These sequences are usually silenced by methylation but are transcribed de novo as a result of the general demethylation of the genome that occurs in cancer cells. This was suggested by the finding that cells deficient in RNaseL were highly resistant to the cytotoxicity of 5-azacytidine, a drug that demethylates the genome. Cells were made resistant to 5-azacytidine via inhibition of RNaseL [11].

Other cancer studies found that allelic variation in RNaseL was observed to be associated with increased risk of prostate cancer and the increased risk of higher tumor grade, together with an increased level of inflammation markers [12, 13]. Allelic variation of RNaseL may modify the risk of breast cancer in patients carrying high risk mutations [14]. The inherited RNaseL alleles responsible for this are proposed to reduce RNaseL activity, and this proposition is supported by observations in lung cancer cells, which increased the levels of the RNaseL inhibitor RL1 to suppress RNaseL-driven apoptosis, with mitochondrial-mediated apoptosis restored by interferon gamma [15].

Our laboratories conducted an *N*-ethyl-*N*-nitrosourea mutagenesis project in mice to find new genes driving mammary gland development and function, with the intent of also identifying new genes involved in mammary cancer initiation and metastasis. We discovered a dominant mutation in *Oas2*, (referred to as MT, wild type *Oas2* referred to as WT) causing a non-conservative amino acid change that resulted in failed lactation despite normal mammary development during pregnancy. These mice were otherwise completely normal [16]. We observed a robust interferon response associated with apoptosis in the mammary glands of our MT mice. When MT was expressed in human breast cancer cells we found that the interferon response depended on the presence of RNaseL and interferon regulatory factor 7 (IRF7), the key effector molecules at the proximal and distal ends of the OAS-RNaseL signaling pathway [16]. This was the first observation of a regulatory pathway linking activation of viral recognition to the control of lactation. The *Oas2* mutant mouse provides a defined and single molecular event that induces an interferon response, with pathological activity only in the mammary gland at the onset of lactation.

The *MMTV-PyMT* model is the most widely used mouse model of breast cancer. PyMT protein [17] acts as a scaffold promoting persistent signaling via binding of SRC family kinases, PI3K, SHC, 14-3-3, PLC-gamma and TAZ. It causes early and multifocal onset of estrogen receptor positive hyperplasia that rapidly progresses through a series of well-defined lesions to estrogen receptor negative invasive carcinoma of the luminal molecular subtype [18]. The model is weakly sensitive [19] or refractory [20] to PD-L1 or PD-1 as monotherapy. Metastasis to lung is frequent and strongly dependent on innate immune cells previously characterized, as macrophages [21, 22] or myeloid-derived suppressor cells (MDSC) [23] of monocytic (G-MDSC) or neutrophil (PMN-MDSC) origin, and now thought of as pathologically activated monocytes and neutrophils [24].

To determine if activation of OAS2 by a future breast cancer therapeutic could provide a new therapeutic strategy, and to distinguish OAS activation from that of TLR3, we combined the *Oas2* mutation with expression of the polyoma middle T oncogene (*PyMT*) driven by the mouse mammary tumor virus promoter (*MMTV)* [25] and asked if this altered the initiation or progression of mammary cancer, and the interaction with checkpoint immunotherapy.

## Methods

### Mice

The *Oas2* colony [16], on an inbred FVBN background, was crossed with the polyoma middle T antigen mouse (*MMTV-PyMT*) also on an FVBN background, a kind gift from Dr. William Muller [25]. The colony was then maintained by breeding compound heterozygous males (MT *Oas2*/WT *Oas2*/*MMTV-PyMT*/non-transgenic) with WT *Oas2*/WT *Oas2* and non-transgenic FVBN females. All animals were housed with food and water ad libitum with a 12-h day/night cycle at 22 °C and 80% relative humidity.

### Timed mating

One or two 7–9-week-old females were housed with single males overnight and monitored daily for a vaginal plug, and pregnancy was confirmed by weight gain. Females were single housed for parturition and pups culled at birth. Mammary gland tumors were measured with calipers throughout pregnancy and the postpartum period. Tumor volume (mm^3^) was calculated as (minimum measurement^2^) * (maximum measurement)/2. Mice were euthanized at specified endpoints, via anesthesia (5% isoflurane, 1 L/min O_2_) followed by cervical dislocation. At autopsy 4th and combined 2nd/3rd glands or tumor weights were recorded.

### PDL-1 antibody treatment in parous mice

Parous mice received 10 µl per gram body weight of a solution of PDL-1 (cat BP0101) or IgG control (cat BP0090) at 1 mg/ml via I.P. injection for six treatments, delivered twice weekly.

### Mammary gland whole mounts and lung collection

Mouse mammary glands, tumors and lungs were harvested at specified timepoints, and fixed in 10% buffered formalin for 4 h at room temperature. Tissues were changed into 70% ethanol prior to embedding in paraffin. Mammary glands were defatted in 3–4 changes of acetone before being dehydrated and stained in carmine alum for whole mount photography. Glands were then passed through a series of graded alcohols and embedded in paraffin for sectioning and immunohistochemistry.

### Immuno-histochemistry

Tissue sections were either stained with hematoxylin and eosin for routine histochemistry or stained with antibodies to the following antigens using immunohistochemistry protocols as detailed in Additional file 1: Table S1. For analysis of lung metastasis, two or more sections at 100 µm intervals were cut for staining.

### Image analysis

Whole mammary gland or lung sections on glass slides were scanned in Aperio ScanScope XT at Katharina Gaus Light Microscopy Facility, and Garvan Microscope Facility, Sydney, Australia, and analyzed by QuPath [26]. A set of multiple reference images was used to train the DAB pixel detection algorithm. Quantification of phospho-Stat positive nuclei used the positive cell detection QuPath algorithm. Regions that contain positive 3,3′-diaminobenzidine (DAB), hematoxylin or no-stain were classified by batch processing and reported as total tissue area (µm^2^), DAB-stained area (µm^2^), non-DAB-stained area (µm^2^) and non-stained area (µm^2^). DAB stain area percentage without lumen non-stained area was calculated as;

$$\begin{aligned} & {\text{PYMT stain area percentage with non-stained area}} \\ & \quad = \left( {\frac{{\text{PYMT stain area}}}{{{\text{PYMT stain area}} + {\text{non - PYMT stain area}}}}} \right)*100 \\ \end{aligned}$$

Kaplan–Meier survival analysis, graph drawing and statistical tests were performed in GraphPad Prism 8.0.2. Strong evidence against the observation being made by chance was accepted at the 90% level.

### Immune cell profiling by flow cytometry

Mouse mammary glands (2/3rd & 4th) were crudely macerated and cells disassociated in 1% hyaluronidase, 37 °C shaking, 20 min. Digestion was neutralized with 2% fetal calf serum (FCS)/phosphate-buffered saline (PBS) prior to centrifugation at 1200 rpm, 4 °C. Cell pellets were resuspended (25 µg/ml DNAse1 in 2% FCS/PBS) and strained through 40 µm mesh and the supernatant concentrated by centrifugation. Cell pellets were resuspended 0.8% NH_4_Cl, DNase1 (25 µg/ml) in PBS and incubated in 37 °C water bath, 1 min. Red blood cells (RBC)/DNA lysis was neutralized in 7 × volume 2% FCS/PBS prior to centrifugation and resuspension in 2% FCS/PBS for immune cell staining using fluorophore conjugated antibodies (refer to antibody panels in Additional file 2: Table S2). Mouse spleen, thymus and lymph nodes (from 4th mammary glands) were collected separately into RPMI media (+ 10% FCS) and strained through 70 µm mesh directly prior to RBC/DNA lysis, cell resuspension and immune cell staining steps (as detailed above). Flow cytometry was performed using BD LSR II SORP and data exported to the FlowJo software (Tree Star Inc.) for data analysis. Routine T [27, 28] and innate immune cell protocols [29] were followed using antibodies listed in Additional file 2: Table S2.

## Results

### Effects of pregnancy and mutation of Oas2 in the MMTV-PyMT model

**Fig. 1:**
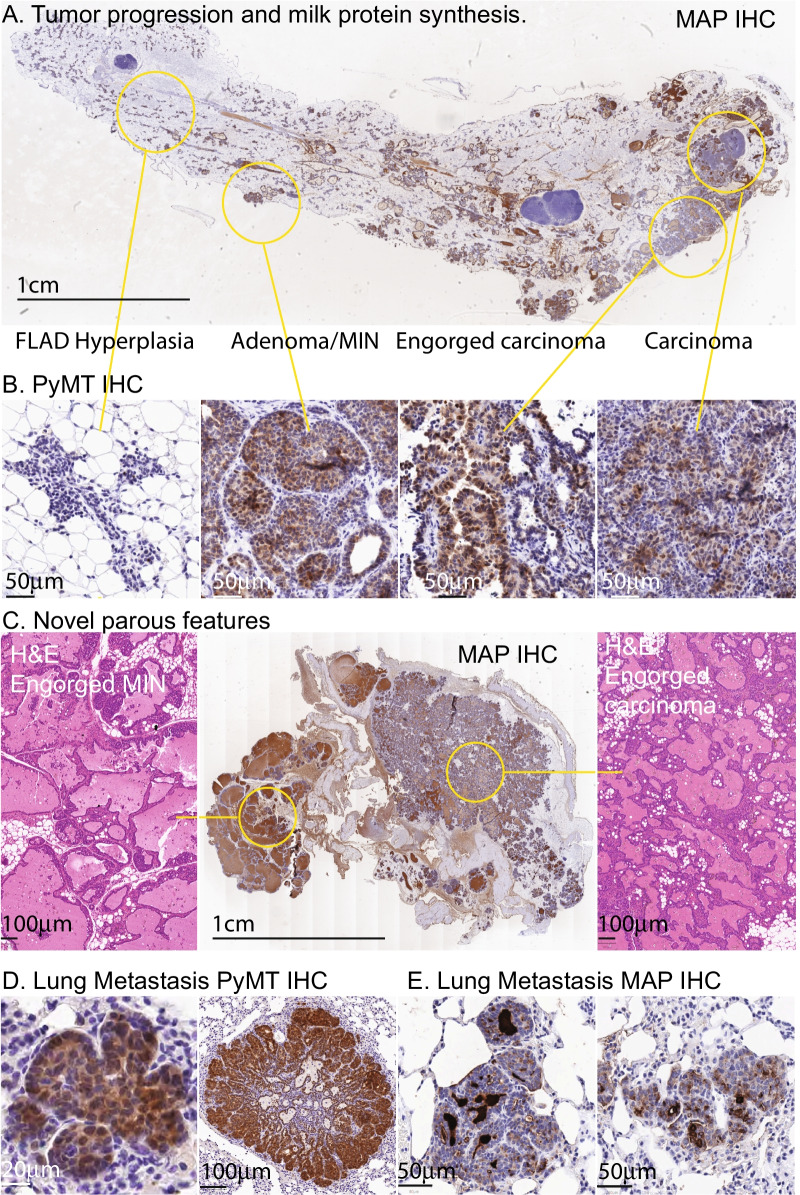
Effects of pregnancy on lesion histopathology. **A** Milk-associated protein (MAP) IHC staining of a number 4 mammary gland at day 3 postpartum from a 9-week-old dam. Inguinal region on the right-hand side, dorsal region on the left-hand side. Lesions identified and named by Lin et al. [18] to be characteristic of disease progression are circled in yellow and are shown at higher magnification below. Failed lobuloalveolar development (FLAD) is indistinguishable from hyperplasia. **B** Corresponding lesion IHC staining for PyMT protein. **C** Novel histopathological features seen in the immediate postpartum period, MAP IHC of a whole 2nd and 3rd mammary glands shown in the center panel and H&E at higher magnification of the circled features shown either side. **D** PyMT staining of metastases, **E** MAP staining of metastases

*MMTV-PyMT* mice were made pregnant at 7–9 weeks of age and analyzed between days 1–3 postpartum. Tumors arose in all glands and were generally first detected by palpation in the combined 2nd and 3rd glands. *MMTV-PyMT* mice showed normal mammary ductal elongation and branching (Fig. 1A), with alveolar bud formation, but failed lobuloalveoli development (FLAD) during pregnancy. At the end of pregnancy, lobuloalveoli would normally completely fill the fat pad and very few adipocytes would be seen, but in *MMTV-PyMT* glands this does not occur. As a result, there were no observable differences in mammary gland development between parous MT and WT mice. The failure of *MMTV-PyMT* dams to feed their pups has been noted previously [25]. This is due to a developmental defect and not physical obstruction of the ductal network as often assumed. The mammary ductal tree showed the same characteristic early lesions identified by Lin et al. [18], hyperplasia, adenoma/MIN and early carcinoma, with late carcinoma dominant at animals that reached the ethical endpoint. These lesions were arranged in a gradient from youngest to oldest epithelium, hyperplasia to carcinoma, left to right in Fig. 1A. IHC showed that PyMT levels were lowest in hyperplasia and highest in adenoma/MIN (Fig. 1B). We observed no histopathological differences between MT and WT mice. Two novel features were observed in parous mice due to the accumulation of milk within the gland (Fig. 1C). In some mice, engorgement with milk of adenoma/MIN produced highly distended ducts, seen on the left-hand side of the combined 2nd and 3rd mammary gland shown in Fig. 1B, which stained strongly for milk-associated protein (MAP). A second feature we have called engorged carcinoma was characterized as misshapen and fluid-filled lumina, formed by a much-thickened fibrous basement membrane containing fibroblasts and immune cells. Epithelial cells accumulated in multiple layers within the lumina. MAP staining was present but weak, suggesting production of the major milk osmoticon lactose, but relative suppression of milk protein synthesis. Areas of early carcinoma and late carcinoma formed within fields of engorged carcinoma, distinguished by the loss of lumina, poorly defined pseudo-alveoli and milk staining limited to developing necrotic areas. Lung metastases showed a uniform pseudo-alveolar pattern typical of the early carcinoma stage. These lesions expanded and developed necrotic centers, but the pseudo-alveolar pattern never varied (Fig. 1D). The metastasis stained strongly for MAP which accumulated within the pseudo-alveoli and in the interstitial spaces (Fig. 1E). Single PyMT positive cells were never seen, the smallest lesions presented as pseudo-alveoli formed from as few as 4 cells, suggesting that metastasis may occur as groups of mammary epithelial cells.

Adenoma/MINs and carcinomas were not engorged with milk at day 15 postpartum or at later times, demonstrating that these features regress dramatically in volume immediately postpartum by reabsorption of the engorging secretions. This is probably responsible for the regressions in tumor volume that we observed by palpation during the postpartum period (Additional file 3: Fig. S1). Tumor regressions and periods of no tumor expansion occurred in 42% and 42% of mutant MT mice, respectively, compared to 21% and 26% of WT mice, respectively. This is consistent with the complete failure of lactation in MT mice [16] and thus faster loss of these engorged features.

**Fig. 2:**
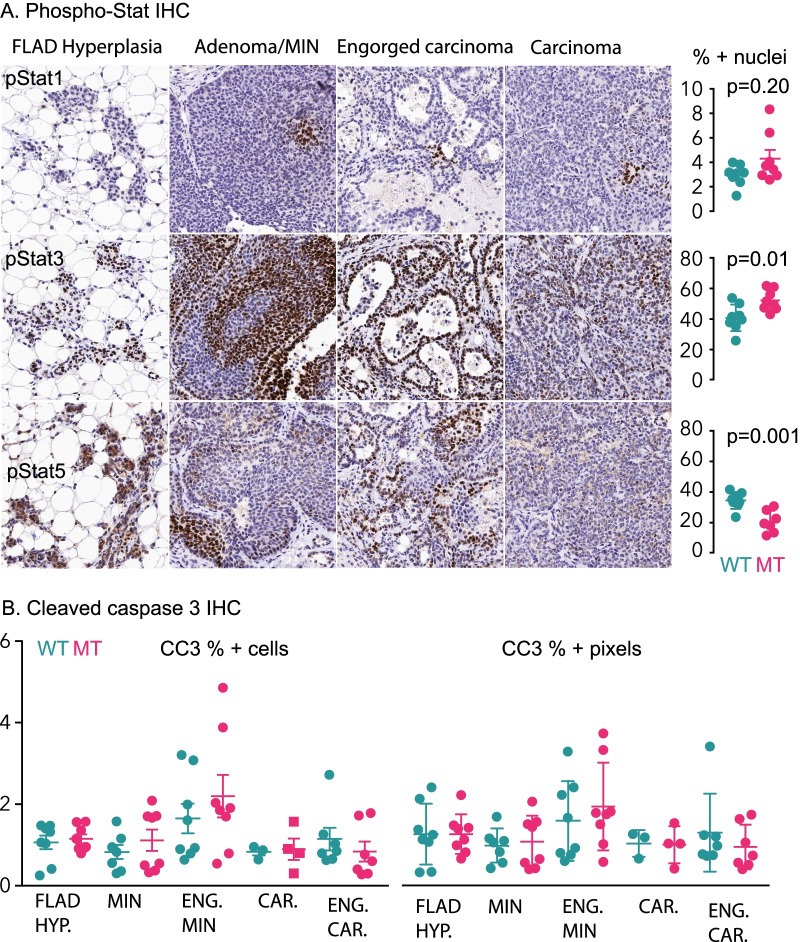
Stat activation and apoptosis during tumor progression in parous mice and the effect of MT *Oas2*. **A** The phosphorylation of the indicated Stat proteins is shown by specific phospho-Stat IHC and DAB staining, within the lesions defined in Fig. 1. QuPath was used to quantify overall mammary gland pStat nuclear positivity in MT and WT glands. Error bars are standard error of the mean, MT Stat1 data is not Gaussian (KS *p* = 0.03) and so the Mann–Whitney *U* test is used to calculate the *p* value. Stat3 and Stat5 *p* values were calculated using Student’s *t* test. **B**. Cleaved caspase 3 (CC3) IHC was used to quantify the proportion of apoptotic cells (CC3% + cells, left hand panel), and the proportion of pixels positive for CC3 staining (CC3% + pixels, right hand panel), in the indicated lesion subtypes abbreviated as HYP.; hyperplasia, MIN; mammary intraepithelial neoplasia, ENG.; engorged, CAR.; carcinoma. All comparisons within lesion subtypes for CC3 between MT and WT are nonsignificant using Student’s *t* test

We previously reported that the *Oas2* mutation caused disruption of Signal Transducer And Activator Of Transcription (Stat) phosphorylation [16] and we determined if this also occurred when combined with the *MMTV-PyMT* oncogene in the immediate postpartum period, when OAS2 levels are highest having been induced through pregnancy [16] (Fig. 2A). Phosphorylated Stat1 (pStat1) was seen as small foci of DAB + nuclei in all stages of tumor progression and quantification using QuPath [26] suggested that some MT animals expressed higher pStat1, as reported in non-*PyMT* animals [16]. Phosphorylated Stat3 (pStat3) was expressed at lower frequency in carcinoma epithelial cells compared to cells from the other stages and more frequently in MT glands. Phosphorylated Stat5 (pStat5) was more frequently expressed in hyperplasia and showed a reduction in the frequency of expression as the disease progressed to carcinoma, correlating with milk protein levels. pStat5 was less frequently expressed in MT glands, as previously reported for non-*PyMT* animals [16]. Thus, the effects of MT on Stat activation continue in *PyMT* animals.

We previously reported increased mammary epithelial cell apoptosis in response to MT in non-PyMT mice following parturition [16]. We used staining for cleaved caspase 3 (CC3) to look for this effect in mice also carrying the *PyMT* oncogene (Fig. 2B). We found no effect when whole glands were analyzed without regard to lesion type prompting an analysis within lesion types. The highest rates of apoptosis were seen in engorged MIN compared to the other lesion types. There were no significant differences in apoptosis between MT and WT animals in any of the lesion types. This suggests that transformation with PyMT abrogates the apoptotic effects of OAS2 activation.

### Effects of MT OAS2 on tumor progression and metastasis

**Fig. 3:**
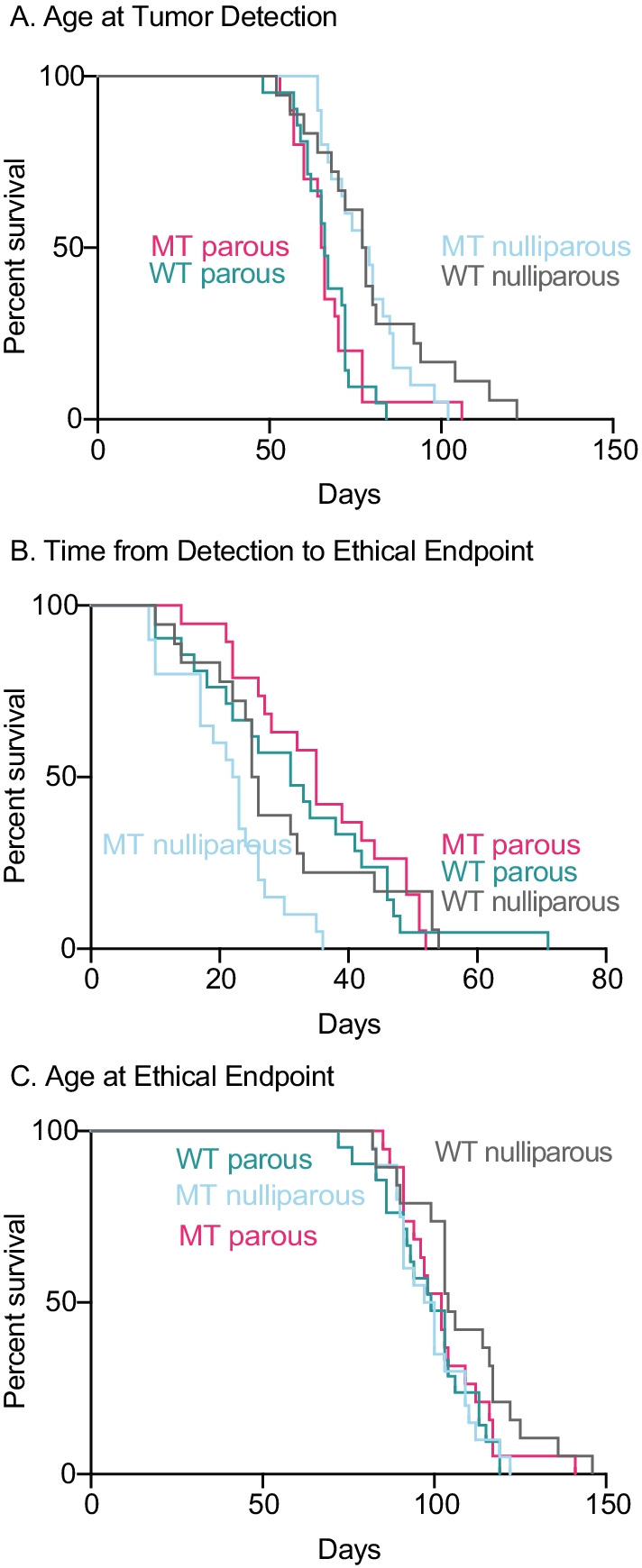
Effect of MT *Oas2* and parity on primary tumor initiation and expansion. Kaplan–Meier survival analysis for the indicated periods and endpoints, and for the indicated genotypes (MT, mutant *Oas2*; WT wild type *Oas2*) and parity status of mouse cohorts (20 per group). Mammary glands were palpated twice weekly and tumor growth was measured, as detailed in Additional file 3: Fig. S1. Two-way Kaplan–Meier curve comparisons and *p* values shown in Additional file 4: Fig. S2, *n* = 19 MT parous, 21 WT parous, 20 MT nulliparous and 19 WT nulliparous.

Cohorts of 20 WT or MT *PyMT Oas2* mice were kept nulliparous or were made pregnant (80 mice total) from 7 to 9 weeks of age and all pups were removed at birth. Mice were monitored by mammary palpation at twice-weekly intervals. Tumor onset and growth was measured in the major and minor axes using calipers, and mice were euthanized at the estimated ethical endpoint of 10% tumor burden prior to autopsy. Animal survival to various endpoints was analyzed using Kaplan–Meier survival plots with log-rank *p* values and hazard ratios calculated by the Cox proportional hazards model as implemented in GraphPad-Prism (Fig. 3, and Additional file 4: Fig. S2 for *p* values and hazard ratios (HR) of two-way comparisons).

Survival to the age of initial tumor detection (Fig. 3A) showed that tumors in WT parous animals were detected at a younger age than WT nulliparous animals, 66 compared to 78 days (*p* = 0.0038). MT parous animals also had tumors detected at a younger age than MT nulliparous animals, 65.6 days compared to 77.5 days (*p* = 0.0019). There was no difference in the age of tumor detection between WT and MT animals, either parous or nulliparous. The period from tumor detection to ethical endpoint (Fig. 3B) was not different among WT nulliparous, WT parous or MT parous animals. MT nulliparous animals progressed more quickly than those in the other cohorts, 22.5 days compared to 31 days (*p* = 0.002) for WT parous, 35 days (*p* < 0.0001) for MT parous and 25.5 days (*p* = 0.03) for WT nulliparous. The age that animals reached their ethical endpoint (Fig. 3C) was not different among WT parous, MT parous and MT nulliparous animals. WT nulliparous reached their ethical endpoint later by 5 days (*p* = 0.0345), 3 days (*p* = 0.034) and 4 days (*p* = 0.18), respectively. Overall, parity caused earlier tumor detection but slower tumor progression, opposing effects with the result that parous animals, either MT or WT, reached the ethical endpoint a few days earlier than nulliparous WT animals. The *Oas2* mutation had no effect on the age of tumor detection but caused more rapid progression in nulliparous animals, so that MT nulliparous animals reached the ethical endpoint earlier than nulliparous WT, and at the same age as parous animals. These effects showed HR between 1.8 and 2.9 indicating a two- to threefold increase in risk.

**Fig. 4:**
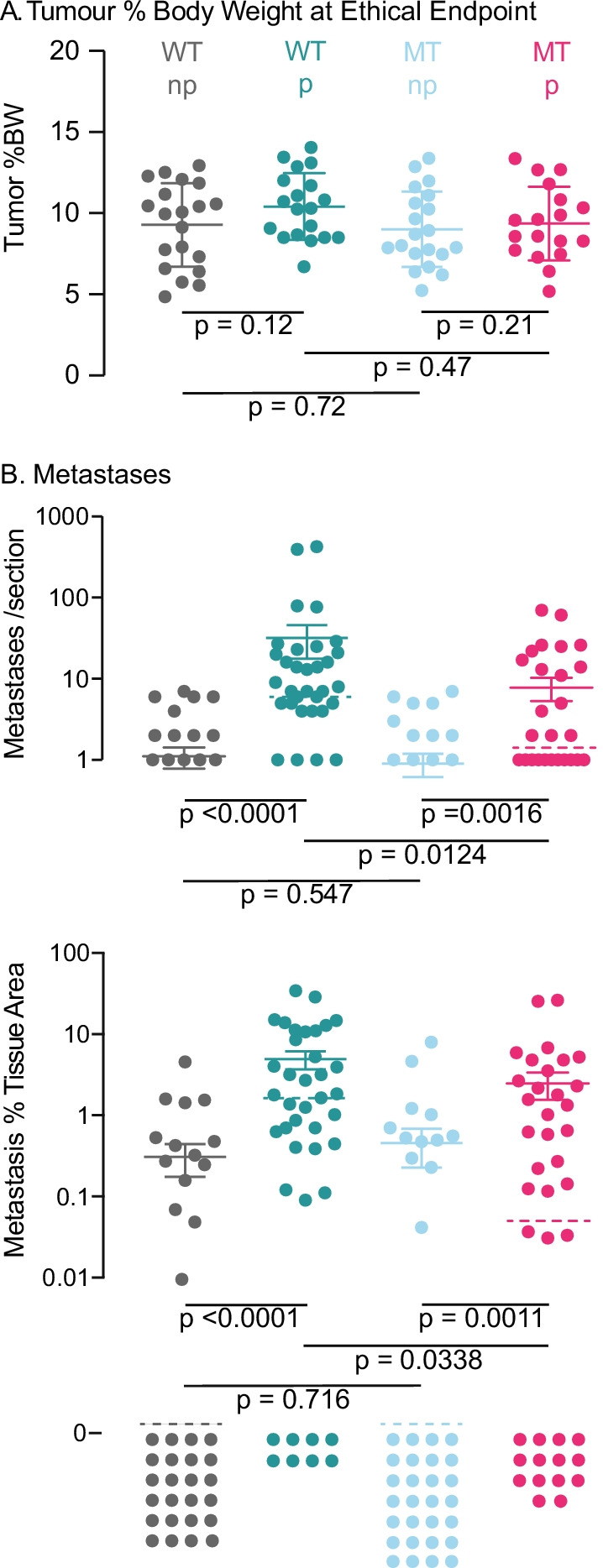
Effect of MT *Oas2* and parity on lung metastases. **A** Actual tumor burden of the cohorts. All tumors were excised and weighed at autopsy, which was initiated when estimates of tumor volume calculated from caliper measurements reached 10% of body weight (BW). Abbreviations are MT, mutant *Oas2*; WT wild type *Oas2*; np, nulliparous; p, parous. Error bars are standard error of the mean and *p* values for the two-way comparisons indicated by the extremities of the lines were calculated using Student’s *t* test. Each symbol is the total tumor burden for a mouse. **B** top panel, the number of lung metastases per section counted manually using two sections per lung, 100 µm apart, and bottom panel, the area of DAB + cells stained by IHC for PyMT and quantified by QuPath, for the indicated genotypes and parity status of the mouse cohorts (20 per group). Error bars are standard error of the mean, median value is indicated by the broken bar, and *p* values were calculated using Mann–Whitney *U* test. Sections without metastases (which cannot be plotted on a log axis) are shown at the bottom of the figure marked “0.” Each symbol is a section

We examined metastatic burden in two sections of both lungs per mouse from the cohort of mice above, using PyMT IHC to detect metastases and allow measurement of their stained area using QuPath [26]. In the cohorts, there was no difference in tumor burden at the ethical endpoint, measured at autopsy by weighing tumors and body weights (BW) from animals euthanized at an estimated 10% tumor burden by tumor dimensions (Fig. 4A, each symbol is a mouse).

Results for the quantification of metastases are plotted on a log scale due to the skewed and thus non-Gaussian distribution of this data, *p* < 0.001 K2 33–70 using the D’Agostino&Pearson normality test (Fig. 4B). Sections with no lung metastases are indicated as “0” at the bottom of the figure as they cannot be plotted on a log axis. Error bars show standard error of the mean, but median values (dashed lines) and the Mann–Whitney nonparametric test of significance are used for comparisons.

Pregnancy caused an increase in the proportion of WT mice with metastases, up from 9 of 19 nulliparous WT mice to 15 of 19 parous WT mice (*p* = 0.09 Fisher’s exact test). In contrast, pregnancy made no difference to the number of MT *Oas2* mice with a metastasis, 10 of 20 nulliparous MT mice compared to 8 of 18 parous MT mice (*p* = 0.8 Fisher’s exact test). There were half the number of MT mice with metastases than WT mice (*p* = 0.04 Fisher’s exact test).

The median number of metastases per lung section (Fig. 4B top panel) rose from 0 in nulliparous WT or MT mice to 6.5 in parous WT mice (*p* < 0.0001 Mann Whitney), but to just 1 in parous MT mice (*p* = 0.0016). Parous MT mice had sevenfold fewer metastases than WT mice (*p* = 0.0124). Image analysis was used to quantify the area of lung metastases as a proportion of lung tissue area (Fig. 4B lower panel). The area of lung alveolar space was excluded from the analysis as individual sections showed variation in the degree of lung inflation at fixation. Parity caused the median area of metastasis in WT lung tissue to increase from 0 to 1.3%, but to just 0.18% in MT animals, a sevenfold decrease in metastases area (*p* = 0.0338). We conclude that activation of OAS2 prevented pregnancy from driving metastases to the lung.

### Effects of the OAS2 mutation on the immune system

Lung metastasis in the *MMTV-PyMT* model is highly dependent on innate immune cells [18, 21–23] of monocyte and neutrophil origin which are recruited to tumors where they adopt a pathogenic inflammatory and immunosuppressive state capable of modulating T-cell activity, often called MDSC of granulocyte or polymorphonucleated neutrophil origin (G- or PMN-MDSC), or monocyte origin (M-MDSC). The development of cell surface markers enabling clear distinction of cells that have assumed the pathogenic state remains the key challenge of this field, and the definition of the pathogenic state is currently limited to functional tests [24].

**Fig. 5:**
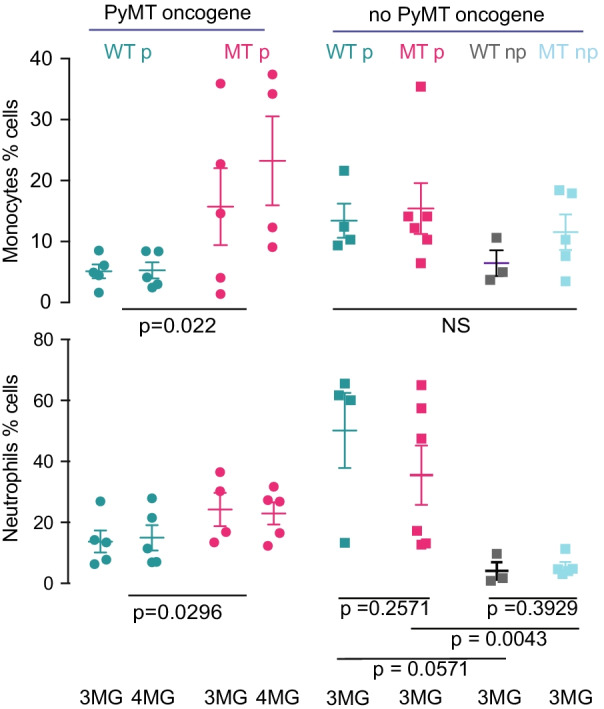
Effects of MT *Oas2* on T cells, neutrophils and granulocytes. A screening of monocytes and neutrophils also known as myeloid-derived suppressor cells (MDSC) was undertaken in mammary tumors. Granulocyte and neutrophil levels were quantified in the third (3MG) or fourth (4MG) and results are combined for statistical analysis. Axes show percentage of cells within the previous gate and gating strategy is shown in Additional file 6: Fig. S4. Error bars are standard error of the mean and *p* values calculated by Student’s *t* test for the two-way comparisons indicated by the extremities of the lines. Abbreviations are MT, mutant *Oas2*; WT wild type *Oas2*; np, nulliparous; p, parous

We undertook a flowcytometric survey of monocytes and granulocytes in tumors (Fig. 5, gating strategy Additional file 6: Fig. S4). Monocyte and neutrophil levels showed individual variation; however, we found a rise in the number of monocytes and neutrophils in more than half of the MT parous tumors compared to WT parous tumors and mean levels increased for both cell types (*p* = 0.022 and *p* = 0.0296, respectively). Non-oncogene carrying mice showed no significant changes in monocyte levels with MT or parity. Neutrophil levels increased significantly in parous animals compared to nulliparous animals (*p* = 0.0571 and *p* = 0.0043) but MT was without effect. The presence of a tumor appeared to reduce the ability of parity to increase neutrophil levels in some mice. As monocyte and neutrophil levels increase in the tumors of parous MT mice, but not in all MT mice, these results suggest that any action of MT on monocytes and neutrophils to reduce metastasis must be to prevent the transition of monocytes and neutrophils to the pathogenic state.

To determine if the changes in monocytes and neutrophils altered parameters measured in CD4 and CD8 T cells, we surveyed tumors, the draining inguinal lymph node, spleen and thymus. We found great variation among individual mice in most of the measured T-cell parameters, showing that we would require an impractically large cohort size to provide sufficient power to this analysis to detect differences (Additional file 5: Fig. S3).

### Effects of PD-L1 on tumor progression and metastasis

To determine if PD-L1 treatment was more effective in parous mice and whether it interacted with MT, we added PD-L1 treatment to the paradigm used above. Mice received either six IgG or PD-L1 treatments (individual details Additional file 6: Fig. S4) when tumors first became palpable and generally coinciding with pregnancy and parity. Mice were monitored with twice weekly measurements of major and minor tumor axis to the ethical end point. Data was analyzed by Kaplan–Meier survival analysis with log-rank *p* values and Cox proportional hazards estimation of hazard ratios. All tumor growth profiles are shown in in Additional file 6: Fig. S4 and all two-way survival comparisons are shown in Additional file 7: Fig. S5.

**Fig. 6:**
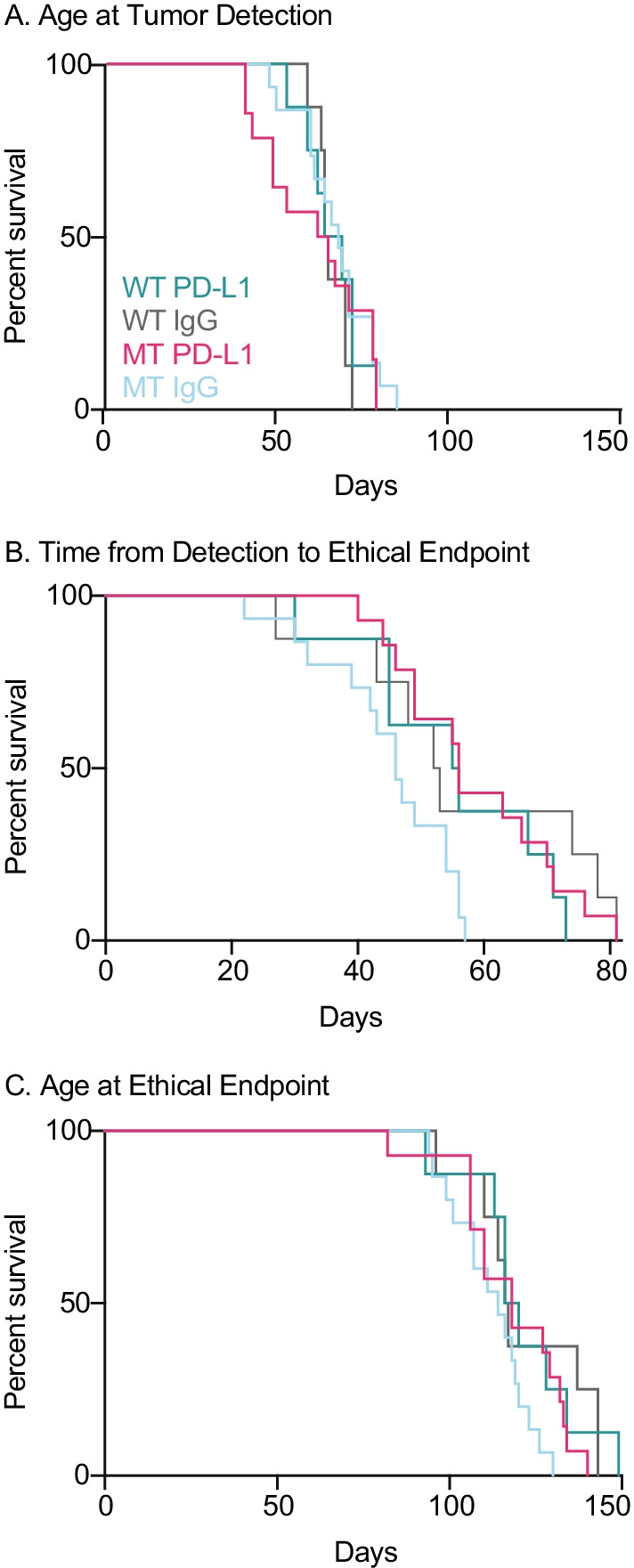
Effect of PD-L1 and MT *Oas2* on primary tumor initiation and expansion in parous mice. Kaplan–Meier survival analysis for the indicated periods and endpoints, and for the indicated genotypes, MT, mutant *Oas2*; WT wild type *Oas2* and treatments (PD-L1 or IgG IP twice weekly), of mouse cohorts (20 per group). All mammary glands were palpated twice weekly and tumor growth was measured by calipers. Individual tumor growth profiles are shown in as shown in Additional file 7: Fig. S5. Two-way Kaplan–Meier comparisons and *p* values are shown in Additional file 8: Fig. S6, *n* = 8 WT PD-L1, 8 WT IgG, 14 MT PD-L1, 15 MT IgG

PD-L1 or MT had no effect on the age at which tumors were first detected (Fig. 6A, two-way comparisons and *p* values Additional file 8: Fig. S6). PD-L1 treatment slowed tumor progression in MT mice from detection to endpoint, by 10 days compared to IgG treated mice (Fig. 6B), from a median time of 46 to 56 days (*p* = 0.0036 HR = 3.8). The effect of PD-L1 in MT mice was also apparent at the ethical endpoint (Fig. 6C), where PD-L1 treated MT mice had a median age of 62.5 days compared to MT IgG treated of 67 days (*p* = 0.065 HR 1.9). PD-L1 had no effect in WT mice. Tumors in MT IgG treated mice progressed more quickly to the ethical endpoint than in WT mice, and PD-L1 treatment normalized this so that MT PD-L1 treated mice became indistinguishable from WT mice. Since PD-L1 was ineffective in WT mice on tumor progression, we conclude that MT sensitized the mice to PD-L1 treatment, but by promoting more rapid tumor progression which was then ameliorated by PD-L1.

**Fig. 7:**
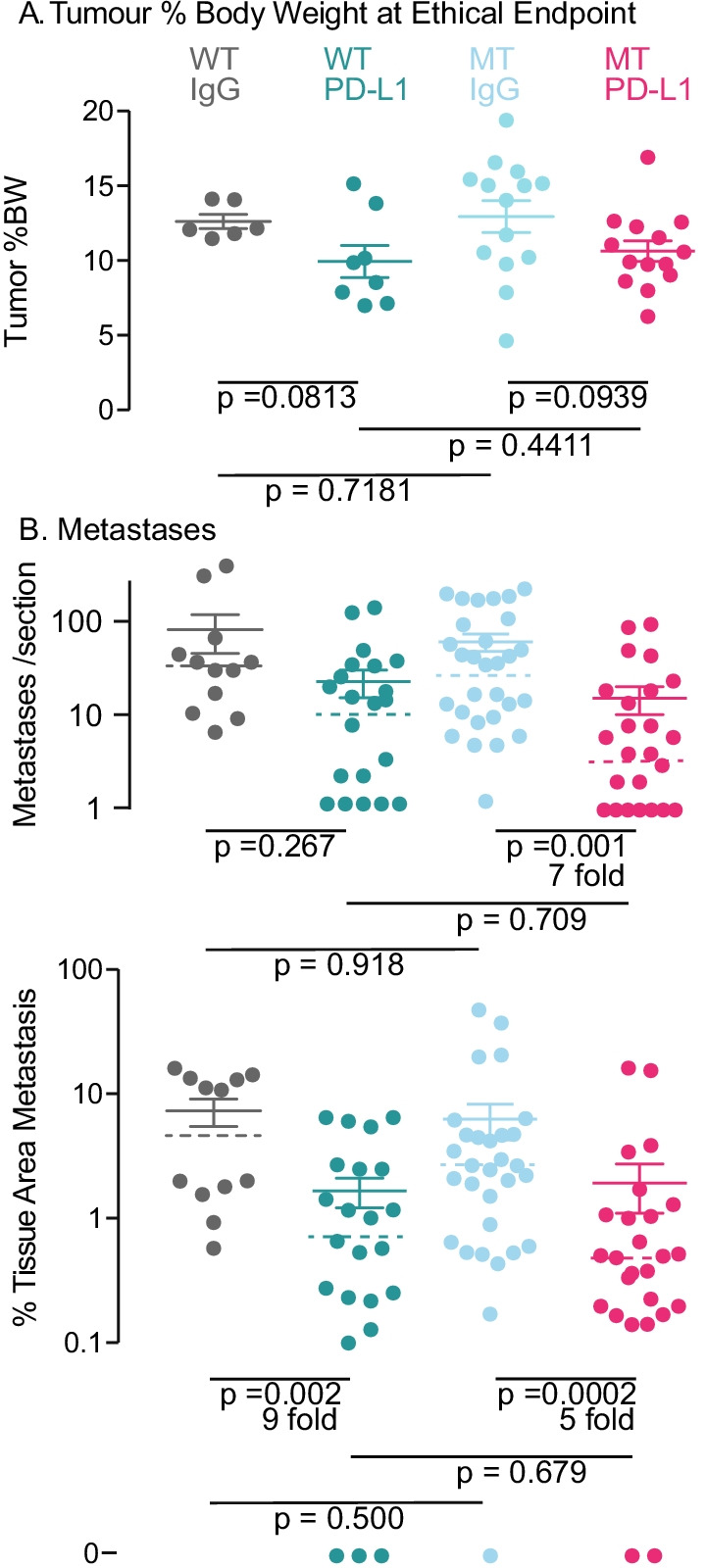
Effect of PD-L1 and MT *Oas2* on lung metastases in parous mice at the ethical endpoint. **A** Actual tumor burden of the cohorts as a % of body weight (BW). All tumors were excised and weighed at autopsy, which was initiated when estimates of tumor volume calculated from caliper measurements reached 10% of body weight. Error bars are standard error of the mean and *p* values were calculated using Student’s *t* test. **B** the number of lung metastases per section, counted manually using two sections per lung 100 µm apart, and the area of DAB + cells stained by IHC for PyMT and quantified by QuPath, for the indicated genotypes and treatments (PD-L1 or IgG IP twice weekly). Mice without metastases (which cannot be plotted on a log axis) are shown at the bottom of the figure marked “0.” Error bars are standard error of the mean, median value is indicated by the broken bar and *p* values were calculated using Mann–Whitney *U* test for the two-way comparisons indicated by the extremities of the line. Abbreviations are MT, mutant *Oas2*; WT wild type *Oas2*

We examined the effect of PD-L1 treatment on metastasis (Fig. 7). Tumor burden was estimated from tumor dimensions and mice were killed at an estimated 10% BW. Actual tumor burden was measured at autopsy. PD-L1 produced slightly lower burden in both WT (*p* = 0.0813) and MT mice (*p* = 0.0939). In WT animals, PD-L1 treatment did not significantly reduce the number of metastases, but in MT animals, a significant sevenfold reduction occurred from a median of 29.5 to 4 metastases per section (*p* = 0.001). The median area of lung metastases as a proportion of lung tissue area was reduced ninefold in WT animals from 6.3 to 0.7 (*p* = 0.002) and fivefold in MT animals from 3 to 0.56 (*p* = 0.0002). We conclude that MT increased the ability of PD-L1 treatment to reduce metastases number, a product of the seeding events to lung, but that no effect was seen in tumor area, a product of seeding and additional metastasis growth. Although the interaction of OAS MT and PD-L1 in this model is of small magnitude, it was detected in a model that is at best partially sensitive to PD-L1, hardly the ideal model to examine this question, but never-the-less provides a finding to be followed in other cancer types.

**Fig. 8:**
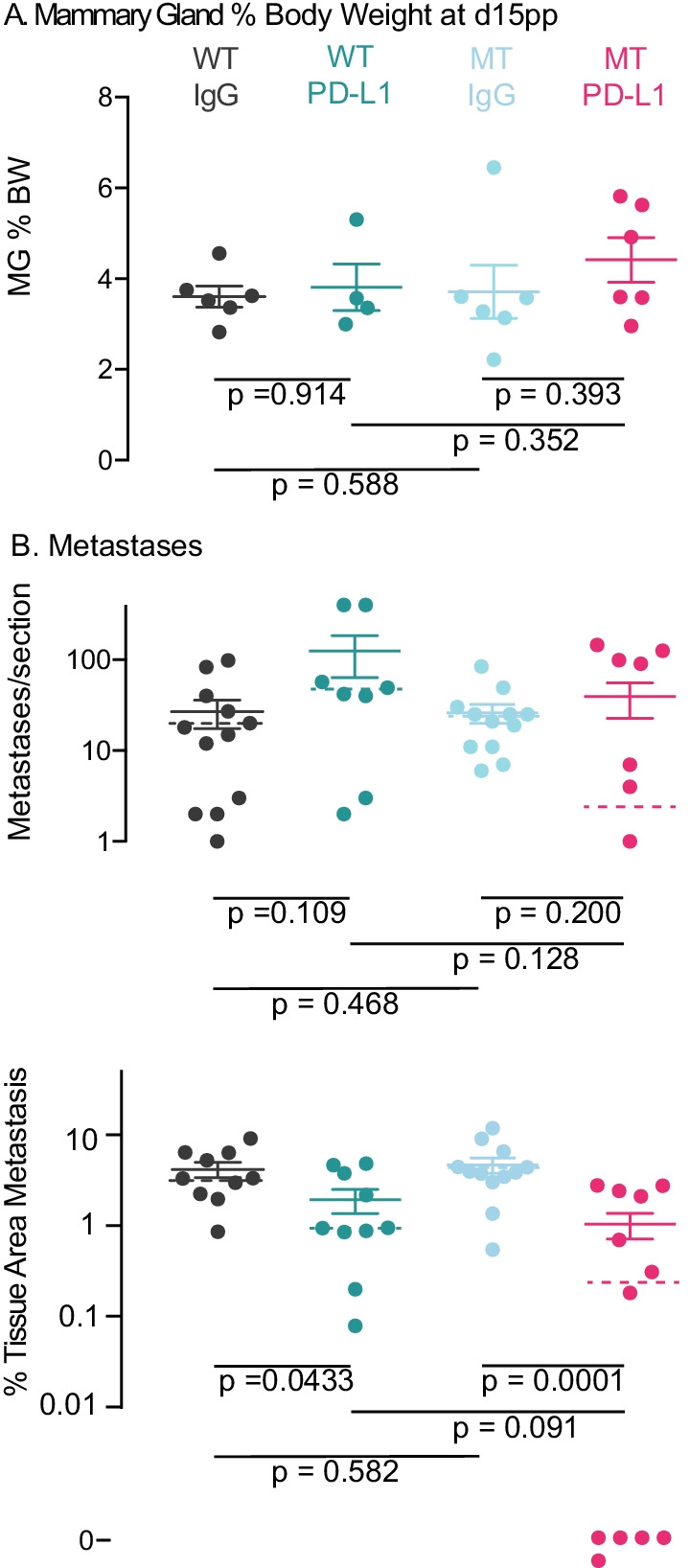
Effect of PD-L1 and MT *Oas2* on lung metastases in parous mice immediately following PD-L1 treatment. **A** Mammary gland (MG) weight as a percentage of body weight (BW). All tumors were excised and weighed at autopsy at the completion of PD-L1 treatment, usually 2 weeks postpartum. Error bars are standard error of the mean and *p* values were calculated using Student’s *t* test. **B** The number of lung metastases per section (two sections per lung) counted manually, and the area of DAB + cells stained by IHC for PyMT and quantified by QuPath, for the indicated genotypes and treatments (PD-L1 or IgG IP twice weekly), of mouse cohorts. Mice without metastases (which cannot be plotted on a log axis) are shown at the bottom of the figure marked “0.” Error bars are standard error of the mean, median value is indicated by the broken bar and *p* values were calculated using Mann–Whitney *U* test. Abbreviations are MT, mutant *Oas2*; WT wild type *Oas2*

To determine if PD-L1 was having a greater but transient effect during the treatment period, we examined a second cohort of six mice per group immediately after treatment using 2 sections per lung (Fig. 8). Mammary gland weights showed no difference among groups (Fig. 8A). The only group in which any lung sections contained no metastases were MT *Oas2* and PD-L1 treated mice, where half the sections were negative (*p* = 0.0373 Fischer’s exact test compared to WT PD-L1 treated). The median number of metastases in WT mice increased 2.8-fold from 16.5 to 45.5, but in MT mice it fell 9.2-fold from 23 to 2.5 (Fig. 8B upper panel). There was a large difference in median metastasis number per section between WT (45.5) and MT (2.5) animals treated with PD-L1 (*p* = 0.128).

Metastases as a percentage of tissue area fell 3.5-fold in WT mice from 3.36% to 0.95% (*p* = 0.0433) and 16-fold in MT mice from 4% to 0.25% (*p* = 0.0001) (Fig. 8B lower panel). PD-L1 was more effective in MT Oas2 mice. Interestingly, the number of metastases seen two weeks after parity was similar to those detected much later at the ethical endpoint, showing that pregnancy and parity increase metastases within an acute period, rather than over the full length of the postpartum period. These data show that PD-L1 treatment caused a reduction of metastases number and area in MT mice.

In summary, MT prevented the increase in the number and the tissue area of lung metastases caused by parity and MT enhanced the response to PD-L1 therapy.

## Discussion

The publications on allelic variation in the Oas-RNaseL pathway suggest that inherited alleles that are thought to cause pathway activation produce better control of virally induced tumors, but also of non-viral tumors by providing an enhanced response to de novo dsRNA production from integrated viruses. A separate group of Oas-RNaseL pathway alleles is thought to cause pathway suppression and to be involved in the genesis of prostate cancer. Our results suggest, however, that the same Oas2 alleles may have context-dependent effects. In the nulliparous state, activation of Oas2 caused faster primary tumor progression, but in the parous state this effect was lost, and instead suppression of metastases and sensitization to checkpoint immunotherapy was observed. It is likely that distinct effects of Oas-RNaseL activation within the cells of the epithelium and immune system add complexity to the role of this pathway in cancer.

A fully completed pregnancy remains an independent predictor of breast cancer risk [30], and pregnancy-associated breast cancer is associated with a 40% increase in mortality due to increased metastasis [30], with even higher rates in cases occurring during pregnancy [31]. The period of increased risk caused by pregnancy may last three or more decades for women who have their first child after their mid 30’s, but less than a decade for women who have their first child before their early 20’s [32, 33]. A degree of life-long protection then follows. This age-dependent period of increased risk defines a large proportion of breast cancer cases as associated with pregnancy; however, current clinical practice uses arbitrary postpartum time periods to define pregnancy associated breast cancer, and does not distinguish pregnancy-associated breast cancer based on the age-dependent period of increased risk. Regardless, this type of breast cancer has no special therapeutic guidelines.

The number of first pregnancies of women over 30 years of age continues to increase especially in developed and developing countries where increasing age at first pregnancy is correlated with increasing female education [34, 35]. Breast cancer associated with pregnancy [36] is one of the main drivers of the increased prevalence of breast cancer [37]. This effect is compounded by an ongoing decrease in breast feeding, as every 12 months decreases breast cancer risk by 4.3%. In developed countries, decreases in childbirth and lactation have doubled the cumulative risk of breast cancer, with two-thirds of this effect attributable to reduced lactation [38].

The results of this study have direct relevance to the changes in reproduction practices in developed and developing nations. Activation of OAS2 in the *MMTV-PyMT* model of breast cancer prevented pregnancy from increasing the number of lung metastases, a finding that establishes the OAS family as therapeutic targets for the development of agents designed to prevent pregnancy from driving breast cancer metastasis. A small interaction with PD-L1 therapy was also detected, albeit in a model at best only partially sensitive to PD-L1. The data provide impetus for further study to determine if the combination of OAS activation and checkpoint inhibition is of therapeutic relevance for the treatment of breast cancer during pregnancy, or other cancers where a more robust effect of PD-L1 occurs. A cautionary finding was that in nulliparous mice activation of OAS2 resulted in more rapid primary tumor progression, suggesting that in nulliparous women the use of interferon-inducing agents should be carefully examined, especially for local recurrences. As the mechanisms responsible for the effects of pregnancy and lactation on postpartum breast cancer risk are poorly understood these findings may also have significance for our understanding of pregnancy associated breast cancer. Importantly, the MT mice show no pathology other than failed lactation, a rare observation in the interferon-response field which is beset with serious adverse events, suggesting that such a therapy would have a favorable toxicity profile.

Our findings may have relevance to breast cancer more generally. We have recently described a pro-metastasis pathology we called involution mimicry [39] that occurs in response to forced expression of the ETS transcription factor ELF5, the master regulator of lobuloalveolar development during pregnancy, which causes a limited lactation and ongoing involution response in an ELF5 mouse model [23], but is also seen in breast cancer patients. The ELF5 mouse model shows a similar a engorgement pathology to that we saw following parity in the MT mice and an increase in lung metastasis that is abrogated by neutrophil depletion [23]. We found profound changes in the state of the epithelium, cancer-associated fibroblasts (CAFs), endothelial cells and immune cells [39]. Among the CAFs we found populations previously associated with mammary gland involution following lactation, and an analysis of ligand-receptor interactions among these cell types established the existence of a complex regulatory network linking the induction of ELF5 in the epithelium to profound transcriptional changes in the CAFs and endothelial cells, and to the recruitment and M2 polarization of MDSC [39]. Two linked human molecular signatures were discovered in breast cancer patients, a lactation signature and an involution signature. The lactation signature was enriched in the epithelial cell populations and the involution signature was present in both epithelial and stromal populations. The involution signature was prognostic in the METABRIC cohort [39]. We surmise that elevated ELF5, possibly due to loss of promoter demethylation [40], drives a limited lactation and involution in these breast cancers.

## Conclusion

Our results raise the possibility that the use of an OAS family activator, or less specific inducers of the interferon response, could reduce the pro-metastases pathology that occurs in a significant number of breast cancers during pregnancy, the immediate postpartum period and in cases expressing the involution-mimicry phenotype, and that efficacy could be increased by combination with checkpoint immunotherapy. While the magnitude of some of the effects detailed here are modest, that they occur indicates the existence of mechanistic links that may be exploited by new therapeutic strategies.

## Supplementary Information

**Additional file 1: Table S1.** Antibodies, concentration and antigen retrieval conditions for immunohistochemistry. All reagents were from Leica BOND or DAKO for automated or manual IHC (as specified). Visualization of antigen–antibody complexes was performed using the DAB + liquid substrate chromogen system (K3467).

**Additional file 2: Table S2.** Antibodies used for flow cytometry. Panel name, antibody name by antigen recognized and conjugated fluorochrome, the antibody supplier and catalogue number and final diluted concentration for each is shown.

**Additional file 3: Fig. S1.** Effect of MT *Oas2* on primary tumor expansion in parous mice. Examples of tumor growth curves of individual mice. The left- and right-hand side 2nd/3rd and 4th mammary glands were palpated twice weekly and tumor growth was estimated by measurement of the major and minor axis of each gland using calipers. Y axis is placed at parity indicated on the x axis as day 0. Numbers show the total number of glands with tumors detected, the number that showed a period of no growth, and the number that showed regression at or following parity. W, wild type; M, MT; W/W, homozygous wild type; M/M, homozygous MT.

**Additional file 4: Fig. S2.** Effect of MT *Oas2* on primary tumor initiation and expansion in nulliparous and parous mice. Two-way Kaplan–Meier survival analysis for the indicated periods and endpoints, and for the indicated genotypes, WT wildtype *Oas2*, MT mutant *Oas2*, and parity status of mouse cohorts (20 per group). *P* values and hazard ratios (HR) calculated by the log-rank test using GraphPad Prism. *n* = 19 MT parous, 21 WT parous, 20 MT nulliparous and 19 WT nulliparous.)

**Additional file 5: Fig. S3.** Effects of MT *Oas2* on T cells in parous mice. A screening of various T-cell parameters was undertaken in mammary tumors, lymph nodes, spleen and thymus. Error bars are standard error of the mean and *p* values calculated by Student’s *t* test (all non-significant). Axes represent % of total cells passing the previous gate. Genotypes, WT/WT homozygous wildtype *Oas2*, MT/MT homozygous mutant *Oas2*.

**Additional file 6: Fig. S4.** Flow cytometry gating strategy for monocytes and neutrophils known as myeloid-derived suppressor cells. Series of gates used to quantify monocytes and neutrophils using flow cytometry.

**Additional file 7: Fig. S5.** Effect of PD-L1 and MT *Oas2* on primary tumor initiation and expansion in parous mice. Examples of tumor growth curves of individual mice. The 2nd/3rd and 4th mammary glands were palpated twice weekly and tumor growth was estimated by measurement of the major and minor axis of individual tumors using calipers. *Y* axis is placed at the day of tumor detection on the *x* axis as day 0. Black diamonds show a treatment with IP IgG or PD-L1 antibodies and red diamonds indicate parity. WT = wild type, MT = MT *Oas2* MT/MT = homozygous MT *Oas2*.

**Additional file 8: Fig. S6.** Effect of PD-L1 and MT *Oas2* on primary tumor initiation and expansion in parous mice. Two-way Kaplan–Meier survival analysis for the indicated periods and endpoints, and for the indicated genotypes and treatments (PD-L1 or IgG IP), of mouse cohorts. P values and hazard ratios (HR) calculated by the log-rank test using GraphPad Prism. *n* = 8 WT PD-L1, 8 WT IgG, 14 MT PD-L1, 15 MT IgG.

## Data Availability

The datasets used and/or analyzed during the current study are available from the corresponding author on reasonable request.

## Abbreviations

BW: Body weight
CAFs: Cancer-associated fibroblasts
CAR: Carcinoma
CC3: Cleaved caspase 3
DAB: 3,3′-Diaminobenzidine
ELF5: E-74 like factor 5
ENG: Engorged
ENU: *N*-Ethyl-*N*-nitrosourea
ETS: E-26 transformation specific
FCS: Fetal calf serum
FLAD: Failed lobuloalveolar development
HR: Hazard ratio
IgG: Immuno gamma globulin
IHC: Immunohistochemistry
IP: Intraperitoneal
IRF7: Interferon regulatory factor 7
MAP: Milk-associated protein
MDSC: Myeloid-derived suppressor cells
MIN: Mammary intraepithelial neoplasia
*MMTV*: Mouse mammary tumor virus promoter
MT: Mutant
*Oas2*: Oligoadenylate synthetase 2 gene
OAS2: Oligoadenylate synthetase 2 protein
PBS: Phosphate-buffered saline
PD-1: Programmed death receptor 1
PD-L1: Programmed death-ligand 1
PI3K: Phosphoinositide 3-kinase
PLC-gamma: Phospho lipase C
polyIC: Polyinosinic–polycytidylic acid
PyMT: Polyoma middle T protein
*PyMT*: Polyoma middle T oncogene
RBC: Red blood cells
RIG1: Retinoic acid induced gene 1
RNase-L: Latent ribonuclease
RPMI: Roswell Park Medical Institute
SHC: Src homology 2 domain-containing
SRC: Rous sarcoma virus family kinases
TAZ: Tafazzin
WT: Wild type
