# Supplementary material for: Activation of the viral sensor oligoadenylate synthetase 2 (Oas2) prevents pregnancy-driven mammary cancer metastases
Source: Breast Cancer Res. 2022 May 3;24:31. doi: 10.1186/s13058-022-01525-z (PMC9066770; doi:10.1186/s13058-022-01525-z)
Supplement: Supplementary file 1 — Additional file 1: Table S1. Antibodies, concentration and antigen retrieval conditions for immunohistochemistry. All reagents were from Leica BOND or DAKO for automated or manual IHC (as specified). Visualization of antigen–antibody complexes was performed using the DAB + liquid substrate chromogen system (K3467). [file 13058_2022_1525_MOESM1_ESM.docx]

**Supplementary Table 1. Antibodies, concentration and antigen retrieval conditions for immunohistochemistry.**

All reagents were from Leica BOND or DAKO for automated or manual IHC (as specified). Visualization of antigen: antibody complexes was performed using the DAB+ liquid Substrate chromogen system (K3467).

| Antigen | Primary Antibody Supplier, Cat # | Primary Ab Concentration & Incubation | Detection System | Antigen Retrieval | Secondary Ab (cat#) |
| --- | --- | --- | --- | --- | --- |
| Polyoma Virus, Middle T Antigen | Novus Biologicals, NB100-2749 | 1:300, 60min | BOND Polymer Refine (DS9800) | HIER 30min, ER2 (EDTA pH9) 100°C | Vector Labs Rabbit anti-Rat (BA-4001-.5) 1:200 |
| Phospho-Stat1 (Y701) | Cell Signalling, 9167 | 1:400, 60min | BOND Polymer Refine (DS9800) | HIER 30min, ER2 (EDTA pH9) 100°C | BOND Rab Polymer only |
| Phospho-Stat3 (Y705) | Cell Signalling, 9145 | 1:400, 60min | BOND Polymer Refine (DS9800) | HIER 30min, ER2 (EDTA pH9) 100°C | BOND Rab Polymer only |
| Phospho-Stat5 (Y964) | Cell Signalling, 9351 | 1:400, 60min | BOND Polymer Refine (DS9800) | HIER 30min, ER2 (EDTA pH9) 100°C | BOND Rab Polymer only |
| Cleaved Caspase 3 | Cell Signalling, 9664 | 1:800, 60min | BOND Polymer Refine (DS9800) | HIER 20min, ER2 (EDTA pH9) 100°C | BOND Rab Polymer only |
| CD8α | Cell Signalling, 98941 | 1:200, 30min | BOND Polymer Refine (DS9800) | HIER 20min, ER2 (EDTA pH9) 100°C | BOND Rab Polymer only |
| Milk Associated Protein | Accurate Chemical & Scientific Co., YNRMTM | 1:12,000, 60min | DAKO manual IHC | DAKO pH6 S1699, 100°C water bath, 20min | Envision Rab (K4009) |
| CD45 | BD Pharmigen, BD553077 | 1:200, 60min | DAKO & Vector Labs manual IHC | DAKO pH6 S1699, 100°C water bath, 20min | VECTASTAIN Elite ABC-HRP (PK-6100) |
