# Supplementary material for: Activation of the viral sensor oligoadenylate synthetase 2 (Oas2) prevents pregnancy-driven mammary cancer metastases
Source: Breast Cancer Res. 2022 May 3;24:31. doi: 10.1186/s13058-022-01525-z (PMC9066770; doi:10.1186/s13058-022-01525-z)
Supplement: Supplementary file 2 — Additional file 2: Table S2. Antibodies used for flow cytometry. Panel name, antibody name by antigen recognized and conjugated fluorochrome, the antibody supplier and catalogue number and final diluted concentration for each is shown. [file 13058_2022_1525_MOESM2_ESM.docx]

**Supplementary Table 2. Antibodies used for flow cytometry.**

Panel name, antibody name by antigen recognised and conjugated fluorochrome, the antibody supplier and catalogue number and final diluted concentration for each is shown.

| Antibody Panel | Antigen & Fluorochrome | Supplier, Cat # | Antibody Concentration |
| --- | --- | --- | --- |
| B & T Cell Panel | CD44 FITC | BD Biosciences,  561859 | 1/300 |
|  | CD366 (Tim-3) PE | BioLegend, 119704 | 1/200 |
|  | CD3 PerCP-Cy5.5 | BioLegend, 100217 | 1/200 |
|  | CD279 (PD-1) PE-Cy7 | eBioscience, 25-9985-82 | 1/400 |
|  | CD25 APC | BD Biosciences,  557192 | 1/100 |
|  | TCR β chain APC -Cy7 | BioLegend, 109220 | 1/400 |
|  | CD44 Pacific blue | BioLegend, 103020 | 1/300 |
|  | Zombie Aqua™ Fixable Viability Kit | BioLegend, 423101 | 1/500 |
|  | CD62L BV605 | BioLegend, 104438 | 1/800 |
|  | CD45R (B220) BV786 | BD Biosciences,  563894 | 1/300 |
|  | CD8a BUV395 | BD Biosciences,  563786 | 1/300 |
|  | CD4 BUV737 | BD Biosciences,  564306 | 1/300 |
| T Cell Panel | CD28 FITC | BioLegend, 122007 | 1/200 |
|  | CD25 PE | eBioscience, 12-0251-83 | 1/200 |
|  | CD62L PerCP-Cy5.5 | BD Biosciences,  560513 | 1/200 |
|  | CD279 (PD-1) PE-Cy7 | eBioscience, 25-9985-82 | 1/400 |
|  | CD152 (CTLA4) APC | eBioscience, 17-1522-82 | 1/100 |
|  | CD278 ICOS APC-Cy7 (e780) | Australian Bioresearch, 313529 | 1/400 |
|  | Foxp3 eFLour450 | eBioscience, 48-5773-80 | 1/100 |
|  | Zombie Aqua™ Fixable Viability Kit | BioLegend, 423101 | 1/500 |
|  | CD44 BV605 | BD Biosciences,  563058 | 1/200 |
|  | TCRb Biotin & BV786 Strepavidin | BD Biosciences,  553169 & 563858 | 1/300 (Primary Ab) |
|  | CD8a BUV395 | BD Biosciences,  563786 | 1/300 |
|  | CD4 BUV737 | BD Biosciences,  564306 | 1/300 |
| Myeloid Panel | Gr1 PE | BioLegend, 108408 | 1/200 |
|  | Ly6C PerCP-Cy5.5 | BioLegend, 128011 | 1/200 |
|  | CD45 PE-Cy7 | BioLegend, 103114 | 1/200 |
|  | F4/80 APC | BioLegend, 123116 | 1/200 |
|  | CD11b APC e780 | eBioscience, 47-0122-82 | 1/200 |
|  | Zombie Aqua™ Fixable Viability Kit | BioLegend, 423101 | 1/500 |
|  | Ly6G BV711 | BD Biosciences,  563979 | 1/200 |
