# Supplementary figures and images for: Activation of the viral sensor oligoadenylate synthetase 2 (Oas2) prevents pregnancy-driven mammary cancer metastases
Source: Breast Cancer Res. 2022 May 3;24:31. doi: 10.1186/s13058-022-01525-z (PMC9066770; doi:10.1186/s13058-022-01525-z)

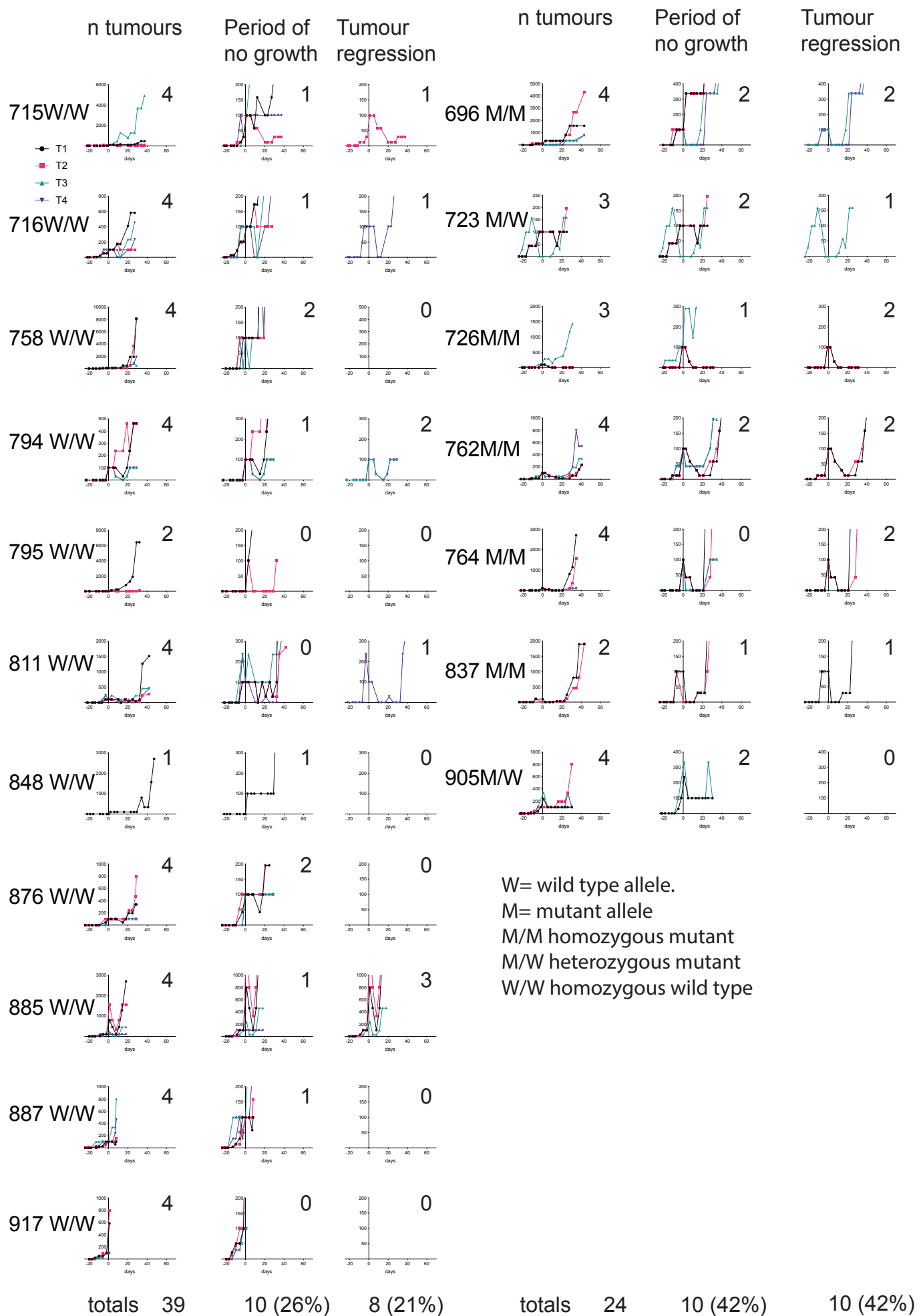

Ho et al. Supplementary Figure 1.

Supplement: Supplementary file 3 — Additional file 3: Fig. S1. Effect of MT Oas2 on primary tumor expansion in parous mice. Examples of tumor growth curves of individual mice. The left- and right-hand side 2nd/3rd and 4th mammary glands were palpated twice weekly and tumor growth was estimated by measurement of the major and minor axis of each gland using calipers. Y axis is placed at parity indicated on the x axis as day 0. Numbers show the total number of glands with tumors detected, the number that showed a period of no growth, and the number that showed regression at or following parity. W, wild type; M, MT; W/W, homozygous wild type; M/M, homozygous MT. [file 13058_2022_1525_MOESM3_ESM.pdf]

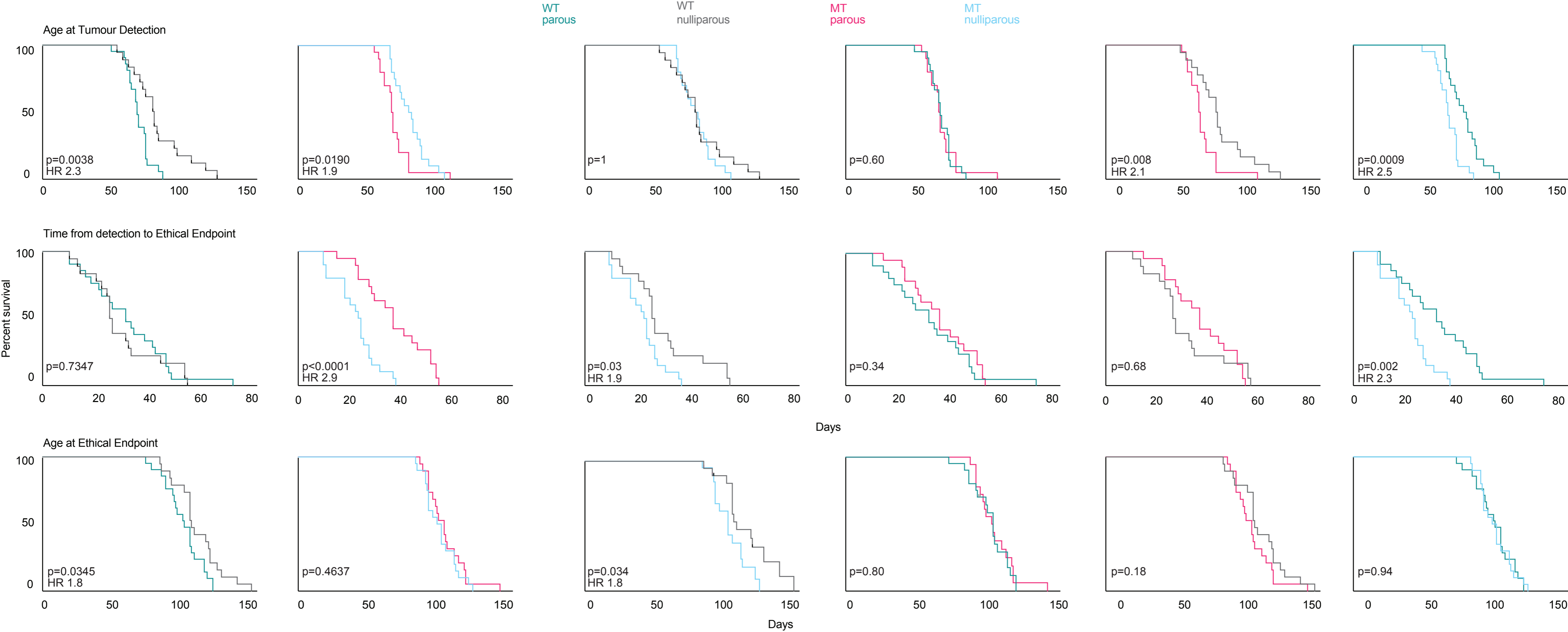

Supplement: Supplementary file 4 — Additional file 4: Fig. S2. Effect of MT Oas2 on primary tumor initiation and expansion in nulliparous and parous mice. Two-way Kaplan–Meier survival analysis for the indicated periods and endpoints, and for the indicated genotypes, WT wildtype Oas2, MT mutant Oas2, and parity status of mouse cohorts (20 per group). P values and hazard ratios (HR) calculated by the log-rank test using GraphPad Prism. n = 19 MT parous, 21 WT parous, 20 MT nulliparous and 19 WT nulliparous.) [file 13058_2022_1525_MOESM4_ESM.pdf]

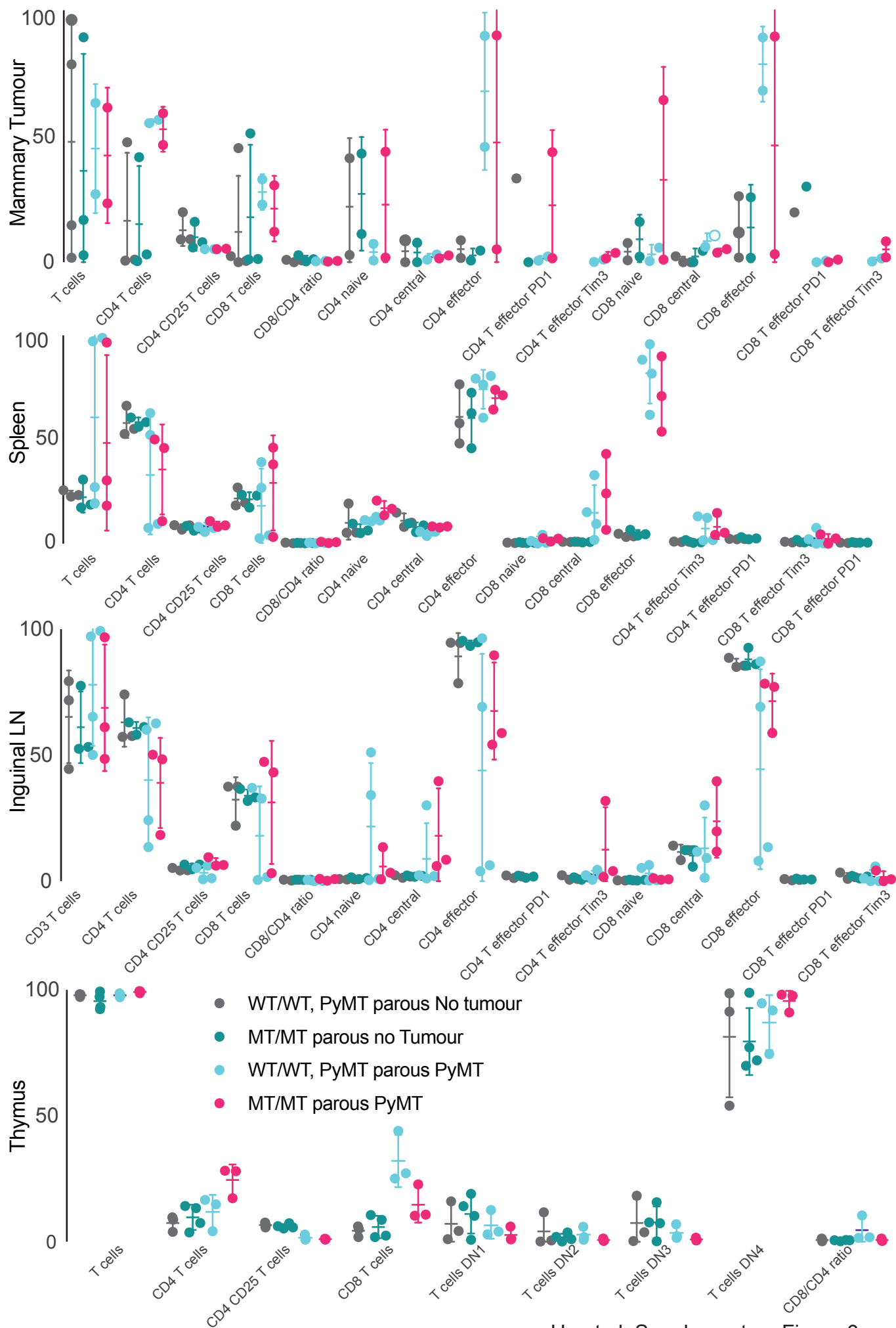

Ho et al. Supplementary Figure 3.

Supplement: Supplementary file 5 — Additional file 5: Fig. S3. Effects of MT Oas2 on T cells in parous mice. A screening of various T-cell parameters was undertaken in mammary tumors, lymph nodes, spleen and thymus. Error bars are standard error of the mean and p values calculated by Student’s t test (all non-significant). Axes represent % of total cells passing the previous gate. Genotypes, WT/WT homozygous wildtype Oas2, MT/MT homozygous mutant Oas2. [file 13058_2022_1525_MOESM5_ESM.pdf]

# Gating strategy monocytes and neutrophils (MDSC)

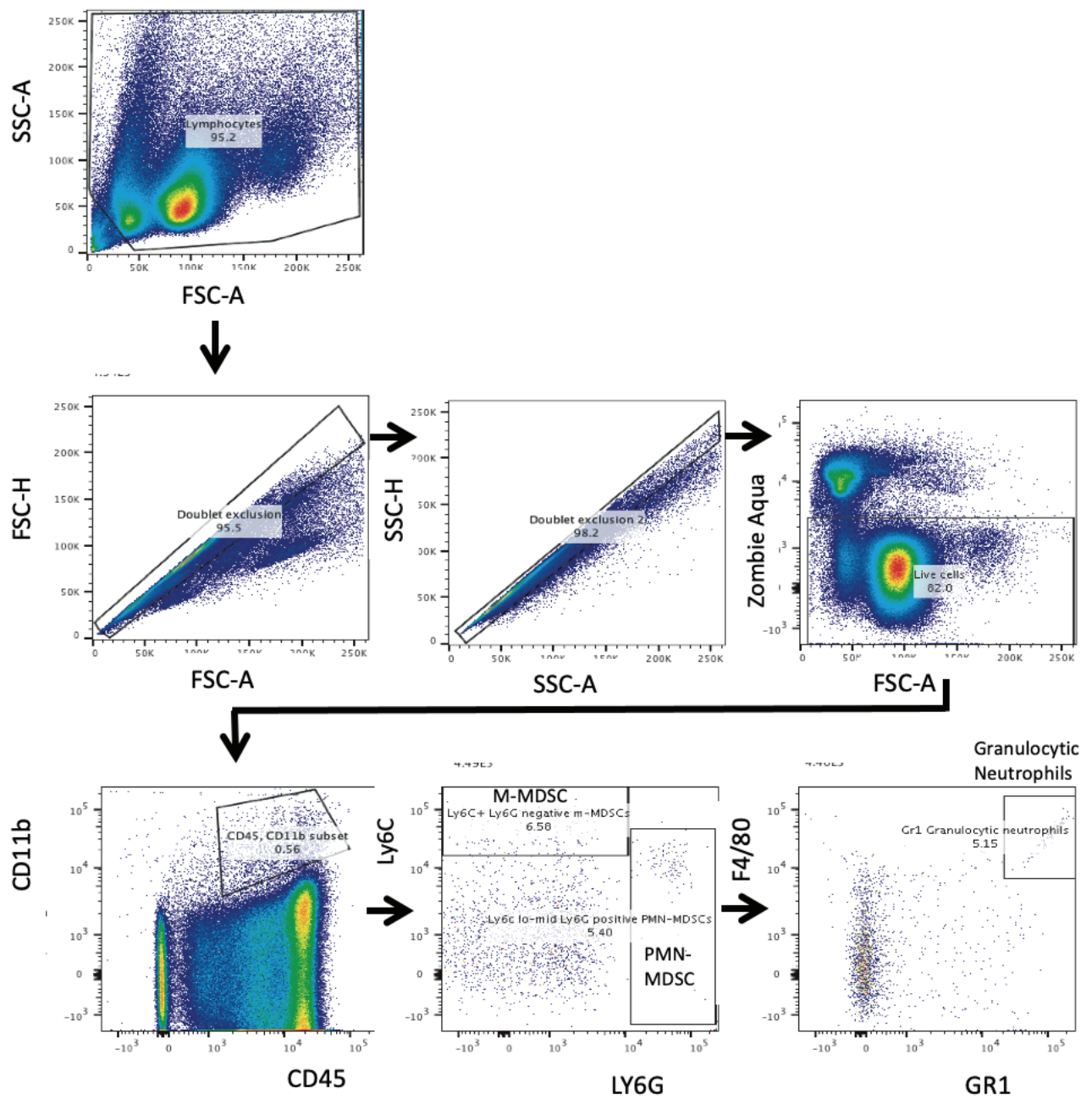

Supplement: Supplementary file 6 — Additional file 6: Fig. S4. Flow cytometry gating strategy for monocytes and neutrophils known as myeloid-derived suppressor cells. Series of gates used to quantify monocytes and neutrophils using flow cytometry. [file 13058_2022_1525_MOESM6_ESM.pdf]

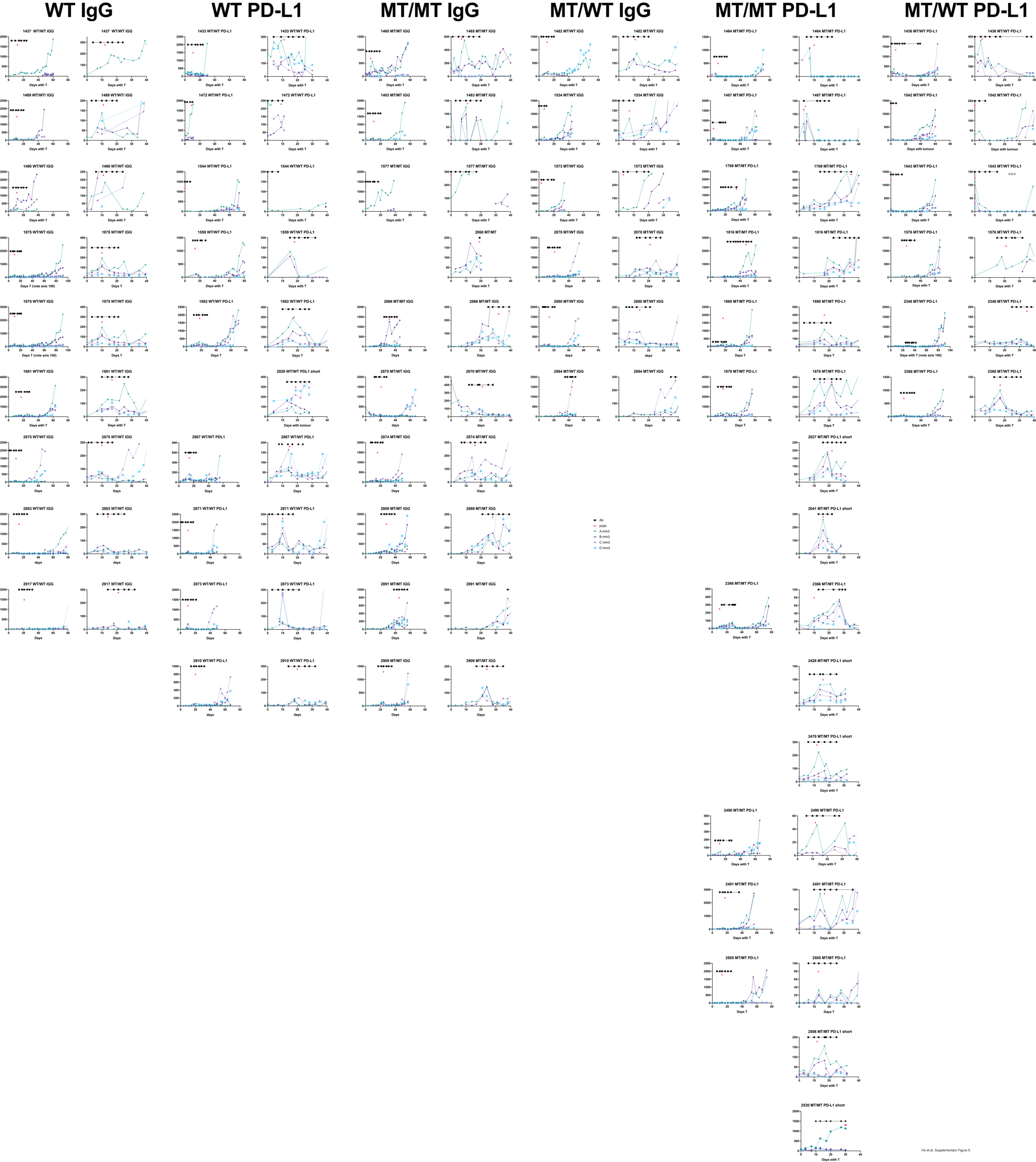

Supplement: Supplementary file 7 — Additional file 7: Fig. S5. Effect of PD-L1 and MT Oas2 on primary tumor initiation and expansion in parous mice. Examples of tumor growth curves of individual mice. The 2nd/3rd and 4th mammary glands were palpated twice weekly and tumor growth was estimated by measurement of the major and minor axis of individual tumors using calipers. Y axis is placed at the day of tumor detection on the x axis as day 0. Black diamonds show a treatment with IP IgG or PD-L1 antibodies and red diamonds indicate parity. WT = wild type, MT = MT Oas2 MT/MT = homozygous MT Oas2. [file 13058_2022_1525_MOESM7_ESM.pdf]

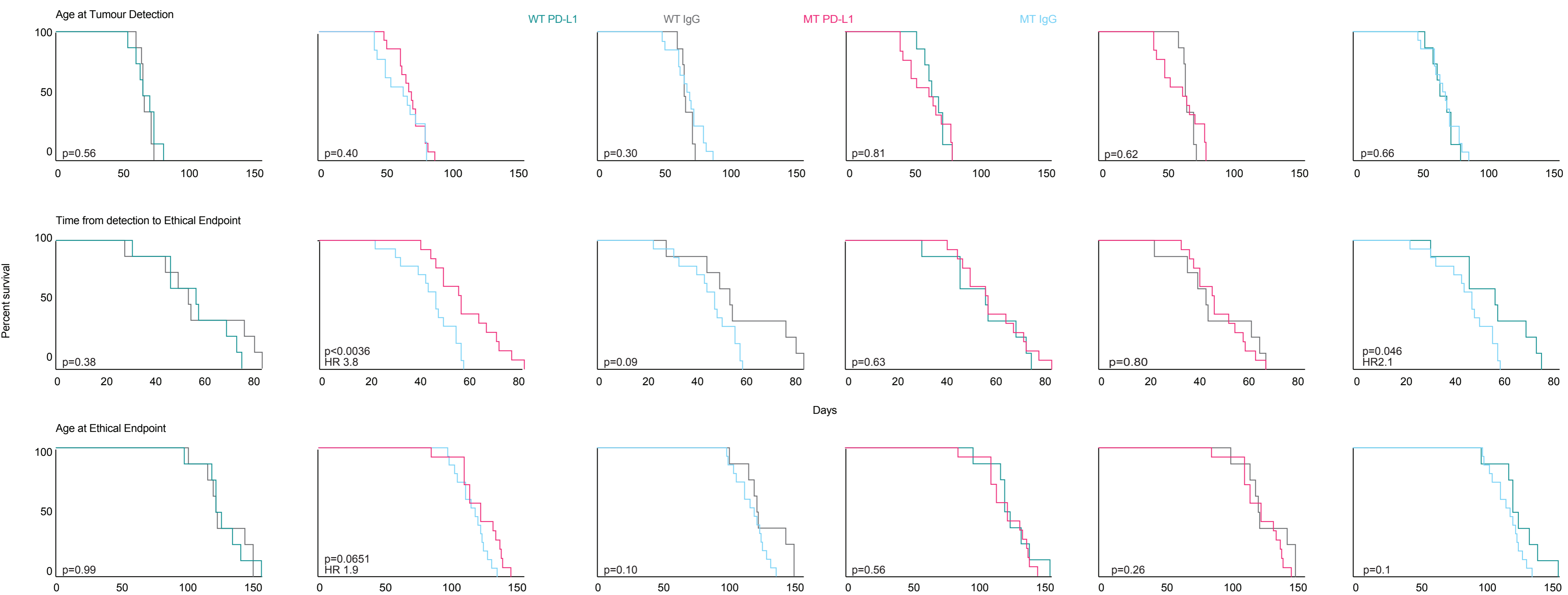

Supplement: Supplementary file 8 — Additional file 8: Fig. S6. Effect of PD-L1 and MT Oas2 on primary tumor initiation and expansion in parous mice. Two-way Kaplan–Meier survival analysis for the indicated periods and endpoints, and for the indicated genotypes and treatments (PD-L1 or IgG IP), of mouse cohorts. P values and hazard ratios (HR) calculated by the log-rank test using GraphPad Prism. n = 8 WT PD-L1, 8 WT IgG, 14 MT PD-L1, 15 MT IgG. [file 13058_2022_1525_MOESM8_ESM.pdf]
